# Clinical Outcomes and Costs of Rivaroxaban for Thromboprophylaxis in Acutely Ill Medical Inpatients: A Cross-Sectional Study

**DOI:** 10.7759/cureus.15497

**Published:** 2021-06-07

**Authors:** Gustavo Lenci Marques, Ana Carolina De Franca, Ana Carolina Saito, Fabiana L Hornung, Ana Carolina Motter, Ana Carolina Falzoni Pontello, Helena Fontana, Juliano Gasparetto, Tiago Zequinão

**Affiliations:** 1 Internal Medicine, Pontifical Catholic University of Parana, Curitiba, BRA

**Keywords:** thromboprophylaxis, acutely ill medical inpatients, cost assessment, rivaroxaban, enoxaparin

## Abstract

Introduction: Venous thromboembolism (VTE) is the primary cause of preventable death in hospitalized patients in the United States. This is a cross-sectional study with a brief cost analysis of thromboprophylaxis with rivaroxaban and enoxaparin in acutely ill medical inpatients.

Methods: The study included a total of 122 patients admitted to a public teaching hospital from December 2019 to January 2021. The sample was equally divided into two groups according to the thromboprophylactic agent prescribed: rivaroxaban or enoxaparin. The primary outcomes included bleeding and symptomatic, ultrasonography-confirmed arterial or venous thrombotic events during or within 90 days after hospitalization. Our secondary outcome was the direct costs of each anticoagulant in US dollars over the 14 months.

Results: During hospitalization, two events were detected in the enoxaparin group: minor bleeding with minimum intervention required (1.6%) and a deep vein thrombosis (DVT) case (1.6%) confirmed by ultrasonography. Within 90 days after discharge, two patients, one of each sample (1.6% vs. 1.6%), were readmitted due to confirmed acute arterial occlusion. Concerning financial assessment, the mean unit cost of enoxaparin during the 14 months assessed was 102.14% more expensive than rivaroxaban.

Conclusions: Both rivaroxaban and enoxaparin showed equivalence in effectiveness and safety in thromboprophylaxis in medical inpatients, aside from possible financial benefit with the first-mentioned drug.

## Introduction

Venous thromboembolism (VTE), a common medical condition worldwide, is the primary cause of preventable death in hospitalized patients in the United States and has an estimated mortality rate ranging from 10% to 30% within 30 days [1-2]. Hospitalization due to acute medical illness, major surgery, prolonged immobilization, active cancer, and advanced age (≥ 75 years) are known risk factors for deep vein thrombosis (DVT) and pulmonary embolism (PE) [3-4]. Current drugs used for thromboprophylaxis in acutely ill medical inpatients include low-molecular-weight heparin (LMWH), unfractionated heparin (UFH), and fondaparinux, as recommended by the American College of Chest Physicians (ACCP) guidelines [5]. However, some disadvantages of these medications, such as parenteral administration and erratic absorption, have encouraged new studies on alternative anticoagulants for thromboprophylaxis in the aforementioned population [6]. Moreover, several studies have shown that other agents, such as rivaroxaban, can be more cost-effective than LMWH in thromboprophylaxis [7].

Rivaroxaban is an oral direct factor Xa inhibitor widely used in the prevention of VTE after major orthopedic surgery. It has been proven to be an effective and safe thromboprophylactic drug when compared to enoxaparin in patients submitted to hip and knee arthroplasty, as well as after below-knee lower-leg fracture surgery [8-11]. Regarding thromboprophylaxis in medical inpatients, the MAGELLAN trial reported noninferiority of rivaroxaban effectiveness and safety when compared to enoxaparin [4].

Until recently, rivaroxaban was used as a thromboprophylactic agent for orthopedic patients only. In 2019, however, this anticoagulant was also approved by the Food and Drug Administration (FDA) for thromboprophylaxis in acutely ill medical patients, even though it is not widely used in these scenarios yet [12-13]. Therefore, our objective in the current study is to evaluate whether rivaroxaban was as safe and effective as enoxaparin in the prevention of VTE in acutely ill medical inpatients. Major risk factors for thrombosis have also been considered. Furthermore, we compared the direct costs of thromboprophylaxis with each anticoagulant.

## Materials and methods

Study design and setting

This is a cross-sectional study with a brief cost analysis of thromboprophylaxis with rivaroxaban and enoxaparin in acutely ill medical inpatients. This study was conducted at Cajuru University Hospital, a 206-bed public teaching hospital specialized in medical and surgical emergencies in the city of Curitiba, Brazil. A total of 122 patients admitted to the internal medicine service from December 2019 to January 2021 were included, equally divided into two groups between oral rivaroxaban, 10 mg daily, and subcutaneous enoxaparin, 40 mg daily. This study was approved by the local ethics committee (Comitê de Ética em Pesquisa da Pontifícia Universidade Católica do Paraná) under approval number 38454620500000020. Informed consent from participants was waived by the Institutional Review Board.

Eligibility criteria

Patients included were above 18 years, who completed anticoagulation with rivaroxaban or enoxaparin for at least two days during hospitalization for acute medical illness, except when DVT/PE was suspected. Patients with a history of continuous anticoagulation therapy or dual antiplatelet therapy were excluded, as well as patients who required intensive care or who underwent surgical procedures due to the primary reason for hospitalization. Patients with active leukemia or metastatic cancer were also excluded. The sample size was determined by the number of patients who used rivaroxaban until the deadline of the study.

Assessment and outcomes

Comorbidities were considered according to the Charlson Comorbidity Index, whereas risk factors for VTE followed those considered in the MAGELLAN trial, which included age ≥ 75 years, acute infectious disease, history of VTE, hormone-replacement therapy, major surgery, or serious trauma within the previous 6-12 weeks and hereditary or acquired thrombophilia. We did not consider body mass index. The primary outcomes included bleeding (classified as minimum, minor, major, or fatal) and symptomatic, ultrasonography-confirmed arterial or venous thrombotic event during or within 90 days after hospitalization. Our secondary outcome was the direct costs of each anticoagulant in US dollars over the 14 months.

Cost analysis

Initially, expenses with each medication were extracted from the hospital pharmacy spreadsheets in the local currency (Brazilian Reals). The average monthly costs of each drug during the period analyzed were calculated and then converted to US dollars according to its average value provided by the Central Bank of Brazil throughout the same period. The average US dollar value corresponded to BRL 5.09, which led us to the average costs of rivaroxaban and enoxaparin per unit. Afterward, these values were multiplied by total days of anticoagulation and the direct mean costs of each thromboprophylactic drug were obtained.

Statistical analysis

Descriptive statistical analysis was performed for all numeric variables. We performed a Student’s t-test or Mann-Whitney test to compare numerical data. For dichotomous data, Fisher’s exact test was used to compare proportions between the two prophylactic agents. All data were tabulated in Microsoft Excel spreadsheets and statistical analysis was performed using Statistical Package for Social Science 21.0 (SPSS 21.0, IBM Corp., Armonk, NY, USA).

## Results

Participants

**Figure 1:**
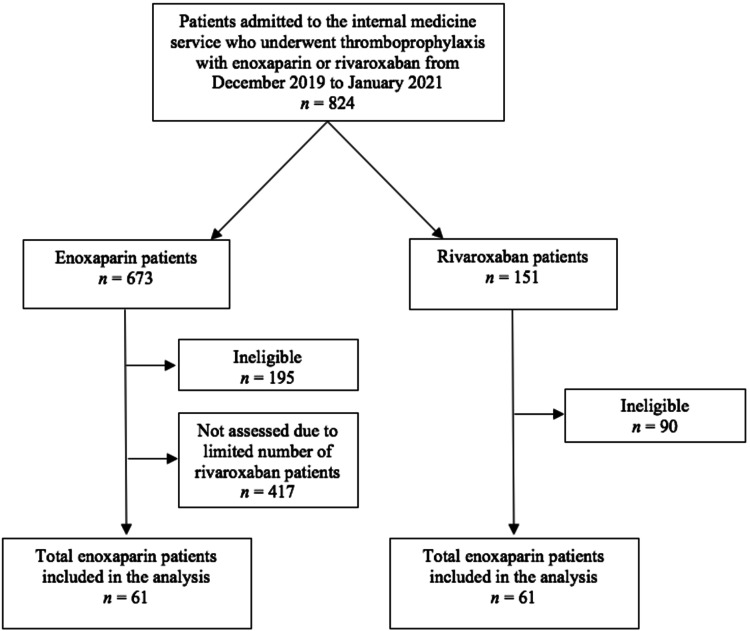
Flow diagram of sample selection.

From December 2019 to January 2021, 824 patients admitted to the internal medicine service used enoxaparin or rivaroxaban for thromboprophylaxis during their admission. A total of 151 patients from the initial sample used rivaroxaban, out of which 61 were eligible. Due to the limited number of patients undergoing prophylaxis with rivaroxaban, only 256 medical records were evaluated from the enoxaparin group, resulting in equal samples, each with 61 patients (Figure 1). Among the analyzed sample, the majority of patients were male (62.3%), with an average age of 60.2 years and a mean hospitalization length of 9.9 days. Other characteristics of the sample included a 1.37 average Charlson Comorbidity Index, prophylaxis extent of 6.9 days, and a total of six deaths in 30 days, from any cause. The main causes of hospitalization were: infectious disease (44.3%); non-surgical trauma (15.6%); abdominal condition (9.0%); metabolic disorder, including electrolyte disturbances, hypoglycemia, wasting syndrome and hypoalbuminemia (7.4%); cardiovascular disease (5.7%); neurological disorder (5.7%); intoxication (4.1%); respiratory disease (4.1%); urinary tract disorder (2.5%); and inflammatory or rheumatic disease (1.6%). Comorbidities and risk factors were described in Table 1.

**Table 1:** Baseline characteristics of patients. SD, standard deviation; IQR, interquartile range; MI, myocardial infarction; AIDS, acquired immunodeficiency syndrome; DVT, deep vein thrombosis; PE, pulmonary embolism *p values ≤ 0.05 †Heart failure patients included were New York Heart Association class III or IV ‡Metabolic disorder included electrolyte disturbances, hypoglycemia, wasting syndrome, and hypoalbuminemia

Characteristic	Enoxaparin (N=61)	Rivaroxaban (N=61)
Mean age (SD) - yr	61.7 (16.9)	58.6 (20.7)
Male sex - no *	32 (52.5%)	44 (72.1%)
Median duration of hospitalization (IQR) - days	8 (5-13.5)	7 (5-11.5)
Median Charlson Comorbidity Index (IQR)	1 (0-2)	1 (0-2)
Median duration of anticoagulation (IQR) – days *	6 (4-10)	4 (3-7.5)
Comorbidity - no	-	-
History of MI	6 (9.8%)	3 (4.9%)
Congestive heart failure	4 (6.6%)	4 (6.6%)
Peripheral vascular disease	1 (1.6%)	3 (4.9%)
Cerebrovascular disease	7 (11.5%)	6 (9.8%)
Dementia	11 (18%)	6 (9.8%)
Chronic pulmonary disease	5 (8.2%)	3 (4.9%)
Rheumatic disease *	6 (9.8%)	0
Peptic ulcer disease	2 (3.3%)	1 (1.6%)
Mild liver disease	4 (6.6%)	2 (3.3%)
Diabetes without end-organ damage	12 (19.7%)	11 (18.0%)
Diabetes with end-organ damage	4 (6.6%)	3 (4.9%)
Moderate to severe renal disease	0	0
Hemiplegia	0	0
Any malignancy without metastases	3 (4.9%)	5 (8.2%)
Leukemia	0	0
Lymphoma	0	0
Metastatic tumor	0	0
Moderate or severe liver disease	0	0
AIDS	3 (4.9%)	2 (3.3%)
Risk factor for DVT - no	-	-
Age ≥ 75 years	13 (21.3%)	13 (21.3%)
History of cancer	3 (4.9%)	5 (8.2%)
History of heart failure †	4 (6.6%)	4 (6.6%)
Acute ischemic stroke with leg paresis	6 (9.8%)	6 (9.8%)
Acute infectious disease	30 (49.2%)	37 (60.7%)
Severe varicosis	1 (1.6%)	1 (1.6%)
History of DVT or PE	0	2 (3.3%)
Hormone-replacement therapy	2 (3.3%)	2 (3.3%)
Major surgery within the previous 6-12 weeks	3 (4.9%)	8 (13.1%)
Serious trauma within the previous 6-12 weeks	10 (16.7%)	9 (14.8%)
Hereditary or acquired thrombophilia	0	0
Chronic venous insufficiency	2 (3.3%)	2 (3.3%)
Death within 30 days - no	4 (6.6%)	2 (3.3%)
Acute medical condition - no	-	-
Abdominal condition	5 (8.2%)	6 (9.8%)
Cardiovascular disease	3 (4.9%)	4 (6.6%)
Infectious disease	28 (45.9%)	26 (42.6%)
Inflammatory or rheumatic disease	1 (1.6%)	1 (1.6%)
Intoxication	3 (4.9%)	2 (3.3%)
Metabolic disorder ‡	4 (6.6%)	5 (8.2%)
Neurological disorder	4 (6.6%)	3 (4.9%)
Non-surgical trauma	7 (11.5%)	12 (19.7%)
Respiratory disease	4 (6.6%)	1 (1.6%)
Urinary tract disorder	2 (3.3%)	1 (1.6%)

Outcome data

Throughout the analyzed period, two events were detected in the enoxaparin group: minor bleeding with minimum intervention required (1.6%) and a DVT case (1.6%) confirmed by ultrasonography. No primary outcome event was registered in the rivaroxaban group during hospitalization. Within 90 days after discharge, two patients, one of each sample (1.6% vs. 1.6%), were readmitted due to confirmed acute arterial occlusion. No significant difference was seen with respect to primary outcomes, as described in Table 2.

**Table 2:** Primary outcomes. p values > 0.05 DVT, deep vein thrombosis

Characteristic	Enoxaparin	Rivaroxaban
DVT during hospitalization	1 (1.6%)	0
Bleeding during hospitalization	1 (1.6%)	0
Thrombotic event within 90 days after hospitalization	1 (1.6%)	1 (1.6%)

Our secondary outcome was to assess the direct mean costs of each medication. Considering the average USD value corresponding to BRL 5.09, the mean unit cost of rivaroxaban was USD 1.40, while the mean unit cost of enoxaparin was USD 2.83. The total expenditure after 14 months in the rivaroxaban group was approximately USD 464.80, which contrasts with a total expenditure of approximately USD 1,443.30 in the enoxaparin group.

## Discussion

The present study assessed the effectiveness and safety of thromboprophylaxis in hospitalized medical patients submitted to anticoagulation with rivaroxaban or enoxaparin. Although limited research regarding thromboprophylaxis has been performed on acutely ill inpatients, our results were similar to those reported in the literature. During an average of 6.9 days of thromboprophylaxis, only minor bleeding and a DVT case were accounted for in the enoxaparin group, whereas no events were observed in the rivaroxaban group. Within the following 90 days, two arterial thrombotic events were reported, one in each group.

Regardless of a similar risk factor prevalence in both samples, more cases of thrombosis and bleeding were reported in the enoxaparin group, but the statistical difference was not significant. The higher incidence of thrombotic events in the enoxaparin group was also described in orthopedic studies, suggesting rivaroxaban may be more effective in thromboprophylaxis [10-11]. As opposed to results found in previous research, however, rivaroxaban was not associated with higher bleeding rates [4]. 

As a secondary outcome, the study analyzed the direct costs of each drug. The mean unit cost of enoxaparin during the 14 months assessed was 102.14% more expensive than rivaroxaban. What this means is that, throughout the same period, thromboprophylaxis with rivaroxaban could have spared USD 978.50. These findings are consistent with a cost-effectiveness study on orthopedic patients that also showed rivaroxaban could be less costly than LMWH [7]. Indirect costs, such as administration and disposal time by the nurse staff, are hypothesized to be higher with enoxaparin thromboprophylaxis, but further studies are required for confirmation. The possibility of thromboprophylaxis for a much lower cost is an important issue, particularly for developing countries, such as Brazil, in which a public national health system takes place.

Our study has some limitations, such as the inherent limitation of the retrospective study design, the size of the sample (restricted by the number of patients in the use of rivaroxaban throughout the analyzed period), and the inclusion of only one hospital. Regarding data collection, patient information was limited to medical records. Therefore, if not specified, it was assumed that patients did not present any additional conditions or take any additional medications. Concerning bleeding assessment, risk factors such as active gastroduodenal ulcer, prior bleeding, low platelet count, hepatic or renal failure, and the presence of a central venous catheter were not considered [14]. Moreover, in accordance with the hospital recommendations, ultrasonography to confirm thrombotic events was performed only in symptomatic patients, which may have underestimated VTE or arterial occlusion outcomes.

Despite the described limitations, the study included a population with broad characteristics, comprising a wide age range. Additionally, the differences observed in the prevalence of comorbidities between the groups did not affect statistics in VTE risk. Therefore, the effectiveness and safety of thromboprophylaxis with rivaroxaban were similar to enoxaparin. A final worth mentioning benefit of thromboprophylaxis with rivaroxaban regards its route of administration. The convenience and safety of oral drug administration, whenever possible, make it a better approach than subcutaneous injection.

## Conclusions

Both rivaroxaban, at a dose of 10 mg once daily, and enoxaparin, at a dose of 40 mg once daily, showed equivalence in effectiveness and safety in thromboprophylaxis in medical inpatients, aside from possible financial benefit with the first-mentioned drug. Further randomized controlled clinical trials are required to support our findings. For future research, the inclusion of a broader number of participants is recommended in order to identify thrombotic events on a larger scale.
